# (*E*,*E*)-4-{4-[3-(4-Chloro­anilino)-1-hydroxy­but-2-enyl­idene]-3-methyl-5-oxo-4,5-dihydro-1*H*-pyrazol-1-yl}­benzene­sulfonamide

**DOI:** 10.1107/S1600536811020617

**Published:** 2011-06-04

**Authors:** Abdullah M. Asiri, Hassan M. Faidallah, Seik Weng Ng

**Affiliations:** a Chemistry Department, Faculty of Science, King Abdul Aziz University, Jeddah 21589, Saudi Arabia; b Department of Chemistry, University of Malaya, 50603 Kuala Lumpur, Malaysia

## Abstract

The mol­ecule of the title compound, C_20_H_19_ClN_4_O_4_S, features a central pyrazole ring that possesses a benzene substituent, as well as a conjugated =C—C=C—C_meth­yl_ substituent. The benzene ring is slightly twisted [dihedral angle = 7.7 (2)°] with respect to the five-membered ring; the mean plane of the zigzag =C—C=C—C fragment [torsion angle = 178.0 (4)°] is also slightly twisted [dihedral angle = 10.6 (4)°]. The amine and hy­droxy groups form intra­molecular hydrogen bonds. The amide group uses one of its H atoms to form a hydrogen bond to the sulfamyl O atom of an inversion-related mol­ecule. Adjacent dimers are further linked by an N—H_amido_⋯N_pyrazole_ hydrogen bond to generate a linear chain. The crystal studied is a nonmerohedral twin with a minor twin component of 25.6 (2)%.

## Related literature

For the synthesis of 4-acetoacetyl-3-methyl-5-onyl-1-phenyl­pyrazole, see: Gelin *et al.* (1983 ▶).
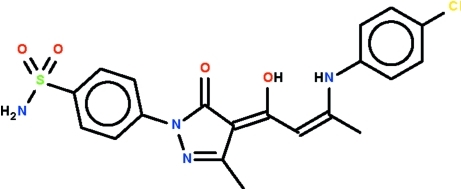


## Experimental

### 

#### Crystal data

C_20_H_19_ClN_4_O_4_S
*M* *_r_* = 446.90
Monoclinic, 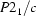

*a* = 14.7513 (17) Å
*b* = 17.545 (2) Å
*c* = 7.6203 (9) Å
β = 101.496 (2)°
*V* = 1932.6 (4) Å^3^
*Z* = 4
Mo *K*α radiation
μ = 0.34 mm^−1^
*T* = 100 K
0.20 × 0.02 × 0.02 mm

#### Data collection

Bruker SMART APEX diffractometer
Absorption correction: multi-scan (TWINABS; Bruker, 2009 ▶) *T*_min_ = 0.935, *T*_max_ = 0.993
32586 measured reflections
3426 independent reflections
2605 reflections with *I* > 2σ(*I*)
*R*_int_ = 0.099

#### Refinement

*R*[*F* ^2^ > 2σ(*F* ^2^)] = 0.054
*wR*(*F* ^2^) = 0.125
*S* = 1.04
3426 reflections
275 parameters
H-atom parameters constrained
Δρ_max_ = 0.39 e Å^−3^
Δρ_min_ = −0.47 e Å^−3^

### 

Data collection: *APEX2* (Bruker, 2009 ▶); cell refinement: *SAINT* (Bruker, 2009 ▶); data reduction: *SAINT*; program(s) used to solve structure: *SHELXS97* (Sheldrick, 2008 ▶); program(s) used to refine structure: *SHELXL97* (Sheldrick, 2008 ▶); molecular graphics: *X-SEED* (Barbour, 2001 ▶); software used to prepare material for publication: *publCIF* (Westrip, 2010 ▶).

## Supplementary Material

Crystal structure: contains datablock(s) global, I. DOI: 10.1107/S1600536811020617/xu5227sup1.cif

Structure factors: contains datablock(s) I. DOI: 10.1107/S1600536811020617/xu5227Isup2.hkl

Supplementary material file. DOI: 10.1107/S1600536811020617/xu5227Isup3.cml

Additional supplementary materials:  crystallographic information; 3D view; checkCIF report

## Figures and Tables

**Table 1:** Hydrogen-bond geometry (Å, °)

*D*—H⋯*A*	*D*—H	H⋯*A*	*D*⋯*A*	*D*—H⋯*A*
O1—H1O⋯O2	0.84	1.79	2.498 (4)	141
N1—H1⋯O1	0.88	2.00	2.659 (4)	131
N1—H1⋯O3^i^	0.88	2.34	3.093 (4)	143
N4—H41⋯N2^ii^	0.88	2.16	3.003 (4)	161
N4—H42⋯O4^iii^	0.88	2.09	2.917 (4)	156

Symmetry codes: (i) 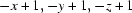; (ii) 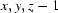; (iii) 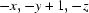.

**Fig. 1.**
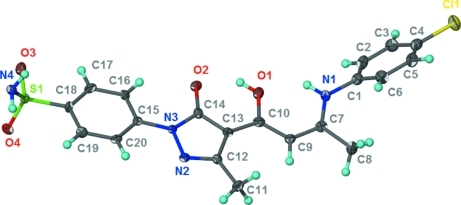
Thermal ellipsoid plot (Barbour, 2001) of C20H19ClN4O4S at the 70% probability level; hydrogen atoms are drawn as spheres of arbitrary radius.

**Fig. 2.**
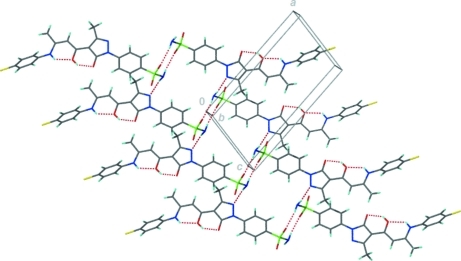
Hydrogen-bonded chain motif.

## supplementary crystallographic information

### Comment

The compound, 4-acetoacetyl-3-methyl-5-onyl-1-phenylpyrazole rearranges under the influence of acetic acid to form functionalized 4-oxopyrano[2,3-*c*]pyrazoles (Gelin *et al.*, 1983). The addition of a sulfamido unit to the phenyl ring is expected to improve its biological activity; in the present study, the *p*-sulfamyl analog is reacted with chloroaniline to yield a new *p*-sulfamylphenylpyrazole derivative (Scheme I). The C_20_H_19_ClN_4_O_4_S (Fig. 1) features a central pyrazole ring that possesses a benzene substituent as well as a conjugated ═ C–C═*C*–C_methyl_ substituent. The benzene ring is slightly twisted with respect to the five-membered ring; the mean plane of the zigzag ═ C–C═ C–C fragment is also slightly twisted.

The amino and hydroxy groups are intramolecular hydrogen-bond donors. The amido group uses one of its H atoms to form an hydrogen bond to the sulfamyl O atom of an inversion-related molecule. Adjacent dimers are further linked by an *N*–H_amido_···N_pyrazole_ hydrogen bond to generate a linear chain motif (Table 2, Fig. 2).

### Experimental

4-Acetoacetyl-3-methyl-5-onyl-1-*p*-sulfamylphenylpyrazole was synthesized by using a literature procedure (Gelin *et al.*, 1983). The compound (1.70 g, 0.005 mol) and 4-chloroaniline (0.63 g, 0.005 mol) were heated in ethanol (25 ml) for 2 h. The solid that separated from solution was collected and recrystallized from ethanol to yield yellow prismatic crystals.

### Refinement

The crystal is a non-merohedral twin with a minor twin domain of 25.6 (2)%. Owing to twinning, the amino, amido and hydroxy H-atoms were generated geometrically.

Carbon-bound H-atoms were placed in calculated positions (C–H 0.95 to 0.98, N–H 0.86, O–H 0.84 Å) and were included in the refinement in the riding model approximation, with *U*(H) set to 1.2 to 1.5*U*_eq_(C,N,O). An *sp*^2^-type of hybridization was assumed for the amino and hydroxy H atoms, and an *sp*^3^-type of hybridization for the amido H atoms.

Omitted because of bad disagreement was (4 4 1).

### Crystal data

**Table:**

C_20_H_19_ClN_4_O_4_S	*F*(000) = 928
*M**_r_* = 446.90	*D*_x_ = 1.536 Mg m^−^^3^
Monoclinic, *P*2_1_/*c*	Mo *K*α radiation, λ = 0.71073 Å
Hall symbol: -P 2ybc	Cell parameters from 656 reflections
*a* = 14.7513 (17) Å	θ = 2.3–20.1°
*b* = 17.545 (2) Å	µ = 0.34 mm^−^^1^
*c* = 7.6203 (9) Å	*T* = 100 K
β = 101.496 (2)°	Prism, yellow
*V* = 1932.6 (4) Å^3^	0.20 × 0.02 × 0.02 mm
*Z* = 4	

### Data collection

**Table:**

Bruker SMART APEX diffractometer	3426 independent reflections
Radiation source: fine-focus sealed tube	2605 reflections with *I* > 2σ(*I*)
graphite	*R*_int_ = 0.099
ω scans	θ_max_ = 25.1°, θ_min_ = 1.4°
Absorption correction: multi-scan (TWINABS; Bruker, 2009)	*h* = −17→17
*T*_min_ = 0.935, *T*_max_ = 0.993	*k* = 0→20
32586 measured reflections	*l* = 0→9

### Refinement

**Table:**

Refinement on *F*^2^	Primary atom site location: structure-invariant direct methods
Least-squares matrix: full	Secondary atom site location: difference Fourier map
*R*[*F*^2^ > 2σ(*F*^2^)] = 0.054	Hydrogen site location: inferred from neighbouring sites
*wR*(*F*^2^) = 0.125	H-atom parameters constrained
*S* = 1.04	*w* = 1/[σ^2^(*F*_o_^2^) + (0.0457*P*)^2^ + 2.8595*P*] where *P* = (*F*_o_^2^ + 2*F*_c_^2^)/3
3426 reflections	(Δ/σ)_max_ = 0.001
275 parameters	Δρ_max_ = 0.39 e Å^−^^3^
0 restraints	Δρ_min_ = −0.47 e Å^−^^3^

### Fractional atomic coordinates and isotropic or equivalent isotropic displacement parameters (Å^2^)

**Table:**

	*x*	*y*	*z*	*U*_iso_*/*U*_eq_	
Cl1	1.06569 (7)	0.72948 (6)	1.73048 (15)	0.0256 (3)	
S1	0.14888 (7)	0.47564 (6)	0.00333 (13)	0.0137 (2)	
O1	0.58091 (18)	0.59404 (16)	1.0470 (4)	0.0200 (7)	
H1O	0.5745	0.5767	0.9424	0.030*	
O2	0.48625 (17)	0.55620 (16)	0.7504 (4)	0.0187 (6)	
O3	0.22069 (18)	0.43897 (16)	−0.0680 (4)	0.0194 (7)	
O4	0.07052 (18)	0.43097 (15)	0.0288 (4)	0.0169 (6)	
N1	0.6886 (2)	0.64430 (18)	1.3466 (4)	0.0147 (7)	
H1	0.6866	0.6177	1.2480	0.018*	
N2	0.2764 (2)	0.63178 (18)	0.8250 (4)	0.0129 (7)	
N3	0.3294 (2)	0.59328 (17)	0.7196 (4)	0.0115 (7)	
N4	0.1079 (2)	0.54401 (18)	−0.1294 (4)	0.0158 (7)	
H41	0.1513	0.5783	−0.1304	0.024*	
H42	0.0610	0.5652	−0.0922	0.024*	
C1	0.7783 (3)	0.6666 (2)	1.4438 (5)	0.0181 (9)	
C2	0.8495 (3)	0.6126 (2)	1.4652 (5)	0.0195 (9)	
H2	0.8370	0.5625	1.4200	0.023*	
C3	0.9374 (3)	0.6319 (2)	1.5512 (5)	0.0200 (9)	
H3	0.9860	0.5955	1.5648	0.024*	
C4	0.9541 (3)	0.7048 (2)	1.6175 (5)	0.0187 (9)	
C5	0.8863 (3)	0.7590 (2)	1.5964 (5)	0.0214 (10)	
H5	0.8998	0.8088	1.6429	0.026*	
C6	0.7972 (3)	0.7406 (2)	1.5065 (6)	0.0205 (9)	
H6	0.7499	0.7782	1.4880	0.025*	
C7	0.6074 (3)	0.6606 (2)	1.3933 (5)	0.0140 (9)	
C8	0.6086 (3)	0.6938 (2)	1.5746 (5)	0.0218 (10)	
H8A	0.6646	0.6771	1.6577	0.033*	
H8B	0.5539	0.6766	1.6182	0.033*	
H8C	0.6081	0.7496	1.5666	0.033*	
C9	0.5228 (3)	0.6492 (2)	1.2803 (5)	0.0139 (8)	
H9	0.4692	0.6617	1.3257	0.017*	
C10	0.5088 (3)	0.6208 (2)	1.1047 (5)	0.0149 (8)	
C11	0.2967 (3)	0.6930 (2)	1.1153 (5)	0.0172 (9)	
H11A	0.2318	0.7065	1.0700	0.026*	
H11B	0.3335	0.7397	1.1420	0.026*	
H11C	0.3018	0.6626	1.2246	0.026*	
C12	0.3320 (3)	0.6480 (2)	0.9771 (5)	0.0118 (8)	
C13	0.4237 (3)	0.6199 (2)	0.9796 (5)	0.0141 (8)	
C14	0.4195 (3)	0.5859 (2)	0.8100 (5)	0.0133 (8)	
C15	0.2875 (2)	0.5682 (2)	0.5466 (5)	0.0113 (8)	
C16	0.3375 (3)	0.5229 (2)	0.4485 (5)	0.0148 (8)	
H16	0.4003	0.5103	0.4957	0.018*	
C17	0.2940 (3)	0.4966 (2)	0.2817 (5)	0.0149 (8)	
H17	0.3275	0.4660	0.2137	0.018*	
C18	0.2025 (3)	0.5143 (2)	0.2129 (5)	0.0136 (8)	
C19	0.1535 (3)	0.5618 (2)	0.3080 (5)	0.0154 (9)	
H19	0.0913	0.5754	0.2589	0.018*	
C20	0.1966 (3)	0.5890 (2)	0.4746 (5)	0.0134 (8)	
H20	0.1641	0.6218	0.5397	0.016*	

### Atomic displacement parameters (Å^2^)

**Table:**

	*U*^11^	*U*^22^	*U*^33^	*U*^12^	*U*^13^	*U*^23^
Cl1	0.0176 (5)	0.0285 (6)	0.0281 (6)	−0.0015 (4)	−0.0013 (4)	−0.0037 (5)
S1	0.0109 (5)	0.0185 (5)	0.0116 (5)	0.0000 (4)	0.0020 (4)	−0.0030 (4)
O1	0.0134 (14)	0.0299 (17)	0.0165 (15)	0.0024 (12)	0.0022 (12)	−0.0079 (13)
O2	0.0117 (14)	0.0284 (16)	0.0166 (15)	0.0036 (12)	0.0045 (12)	−0.0071 (12)
O3	0.0152 (14)	0.0258 (16)	0.0168 (15)	0.0035 (12)	0.0022 (12)	−0.0089 (12)
O4	0.0136 (14)	0.0178 (15)	0.0196 (15)	−0.0041 (12)	0.0036 (12)	−0.0012 (12)
N1	0.0106 (16)	0.0211 (18)	0.0110 (17)	0.0011 (14)	−0.0017 (13)	0.0021 (14)
N2	0.0121 (17)	0.0146 (18)	0.0129 (17)	0.0005 (14)	0.0045 (14)	−0.0004 (13)
N3	0.0080 (16)	0.0151 (17)	0.0119 (17)	0.0014 (13)	0.0032 (13)	−0.0041 (13)
N4	0.0108 (16)	0.0227 (19)	0.0133 (17)	−0.0015 (14)	0.0012 (14)	0.0002 (14)
C1	0.018 (2)	0.022 (2)	0.013 (2)	−0.0011 (18)	0.0024 (17)	0.0011 (17)
C2	0.027 (2)	0.016 (2)	0.014 (2)	0.0011 (18)	0.0015 (18)	−0.0009 (17)
C3	0.018 (2)	0.022 (2)	0.020 (2)	0.0026 (18)	0.0022 (19)	0.0029 (18)
C4	0.019 (2)	0.023 (2)	0.014 (2)	0.0006 (18)	0.0035 (17)	0.0010 (17)
C5	0.024 (2)	0.020 (2)	0.020 (2)	−0.0020 (19)	0.0022 (18)	−0.0028 (18)
C6	0.020 (2)	0.018 (2)	0.023 (2)	0.0012 (19)	0.0032 (18)	0.0014 (18)
C7	0.017 (2)	0.010 (2)	0.015 (2)	0.0028 (16)	0.0034 (17)	0.0035 (16)
C8	0.022 (2)	0.026 (2)	0.017 (2)	−0.0044 (19)	0.0022 (18)	−0.0032 (18)
C9	0.0119 (19)	0.018 (2)	0.013 (2)	0.0014 (16)	0.0033 (16)	0.0000 (16)
C10	0.014 (2)	0.014 (2)	0.017 (2)	0.0003 (17)	0.0055 (17)	−0.0001 (17)
C11	0.017 (2)	0.022 (2)	0.014 (2)	0.0027 (18)	0.0042 (17)	−0.0014 (17)
C12	0.013 (2)	0.0102 (19)	0.013 (2)	−0.0018 (16)	0.0026 (16)	0.0002 (15)
C13	0.0119 (19)	0.016 (2)	0.015 (2)	−0.0017 (16)	0.0040 (17)	−0.0009 (16)
C14	0.0120 (19)	0.014 (2)	0.013 (2)	−0.0008 (16)	0.0006 (16)	−0.0002 (16)
C15	0.0139 (19)	0.011 (2)	0.0101 (19)	−0.0040 (15)	0.0037 (16)	0.0020 (15)
C16	0.0108 (19)	0.019 (2)	0.014 (2)	−0.0025 (17)	0.0016 (16)	−0.0009 (17)
C17	0.0153 (19)	0.018 (2)	0.013 (2)	0.0041 (17)	0.0055 (17)	0.0008 (16)
C18	0.015 (2)	0.015 (2)	0.0098 (19)	−0.0024 (17)	0.0006 (16)	−0.0030 (16)
C19	0.0121 (19)	0.018 (2)	0.016 (2)	0.0027 (17)	0.0023 (16)	0.0000 (17)
C20	0.013 (2)	0.015 (2)	0.014 (2)	0.0018 (16)	0.0064 (17)	−0.0009 (16)

### Geometric parameters (Å, °)

**Table:**

Cl1—C4	1.753 (4)	C6—H6	0.9500
S1—O3	1.436 (3)	C7—C9	1.382 (5)
S1—O4	1.441 (3)	C7—C8	1.497 (5)
S1—N4	1.607 (3)	C8—H8A	0.9800
S1—C18	1.770 (4)	C8—H8B	0.9800
O1—C10	1.316 (5)	C8—H8C	0.9800
O1—H1O	0.8400	C9—C10	1.404 (5)
O2—C14	1.275 (5)	C9—H9	0.9500
N1—C7	1.346 (5)	C10—C13	1.418 (5)
N1—C1	1.435 (5)	C11—C12	1.491 (5)
N1—H1	0.8800	C11—H11A	0.9800
N2—C12	1.310 (5)	C11—H11B	0.9800
N2—N3	1.401 (4)	C11—H11C	0.9800
N3—C14	1.376 (5)	C12—C13	1.435 (5)
N3—C15	1.410 (5)	C13—C14	1.414 (5)
N4—H41	0.8800	C15—C20	1.392 (5)
N4—H42	0.8800	C15—C16	1.398 (5)
C1—C6	1.393 (6)	C16—C17	1.383 (5)
C1—C2	1.399 (6)	C16—H16	0.9500
C2—C3	1.374 (6)	C17—C18	1.382 (5)
C2—H2	0.9500	C17—H17	0.9500
C3—C4	1.379 (6)	C18—C19	1.397 (5)
C3—H3	0.9500	C19—C20	1.387 (5)
C4—C5	1.365 (6)	C19—H19	0.9500
C5—C6	1.393 (5)	C20—H20	0.9500
C5—H5	0.9500		
			
O3—S1—O4	118.65 (17)	H8A—C8—H8C	109.5
O3—S1—N4	108.04 (17)	H8B—C8—H8C	109.5
O4—S1—N4	106.22 (17)	C7—C9—C10	126.0 (4)
O3—S1—C18	106.40 (17)	C7—C9—H9	117.0
O4—S1—C18	108.26 (17)	C10—C9—H9	117.0
N4—S1—C18	109.02 (17)	O1—C10—C9	118.1 (3)
C10—O1—H1O	120.0	O1—C10—C13	116.0 (3)
C7—N1—C1	125.6 (3)	C9—C10—C13	126.0 (4)
C7—N1—H1	117.2	C12—C11—H11A	109.5
C1—N1—H1	117.2	C12—C11—H11B	109.5
C12—N2—N3	106.9 (3)	H11A—C11—H11B	109.5
C14—N3—N2	110.6 (3)	C12—C11—H11C	109.5
C14—N3—C15	129.7 (3)	H11A—C11—H11C	109.5
N2—N3—C15	119.8 (3)	H11B—C11—H11C	109.5
S1—N4—H41	109.5	N2—C12—C13	111.0 (3)
S1—N4—H42	109.5	N2—C12—C11	119.7 (3)
H41—N4—H42	109.5	C13—C12—C11	129.2 (3)
C6—C1—C2	119.7 (4)	C14—C13—C10	119.4 (3)
C6—C1—N1	122.2 (4)	C14—C13—C12	105.3 (3)
C2—C1—N1	118.0 (4)	C10—C13—C12	135.3 (4)
C3—C2—C1	120.3 (4)	O2—C14—N3	126.7 (3)
C3—C2—H2	119.9	O2—C14—C13	127.0 (3)
C1—C2—H2	119.9	N3—C14—C13	106.2 (3)
C2—C3—C4	119.1 (4)	C20—C15—C16	120.5 (3)
C2—C3—H3	120.5	C20—C15—N3	119.6 (3)
C4—C3—H3	120.5	C16—C15—N3	119.9 (3)
C5—C4—C3	121.9 (4)	C17—C16—C15	118.9 (4)
C5—C4—Cl1	118.7 (3)	C17—C16—H16	120.5
C3—C4—Cl1	119.4 (3)	C15—C16—H16	120.5
C4—C5—C6	119.6 (4)	C18—C17—C16	120.8 (4)
C4—C5—H5	120.2	C18—C17—H17	119.6
C6—C5—H5	120.2	C16—C17—H17	119.6
C1—C6—C5	119.3 (4)	C17—C18—C19	120.3 (3)
C1—C6—H6	120.3	C17—C18—S1	118.9 (3)
C5—C6—H6	120.3	C19—C18—S1	120.8 (3)
N1—C7—C9	123.0 (4)	C20—C19—C18	119.3 (4)
N1—C7—C8	118.6 (3)	C20—C19—H19	120.3
C9—C7—C8	118.3 (3)	C18—C19—H19	120.3
C7—C8—H8A	109.5	C19—C20—C15	120.0 (4)
C7—C8—H8B	109.5	C19—C20—H20	120.0
H8A—C8—H8B	109.5	C15—C20—H20	120.0
C7—C8—H8C	109.5		
			
C12—N2—N3—C14	0.0 (4)	C11—C12—C13—C10	2.9 (8)
C12—N2—N3—C15	179.8 (3)	N2—N3—C14—O2	−177.4 (4)
C7—N1—C1—C6	−46.9 (6)	C15—N3—C14—O2	2.8 (7)
C7—N1—C1—C2	137.2 (4)	N2—N3—C14—C13	0.8 (4)
C6—C1—C2—C3	1.5 (6)	C15—N3—C14—C13	−179.0 (4)
N1—C1—C2—C3	177.5 (4)	C10—C13—C14—O2	−2.1 (6)
C1—C2—C3—C4	0.6 (6)	C12—C13—C14—O2	177.0 (4)
C2—C3—C4—C5	−1.6 (6)	C10—C13—C14—N3	179.7 (3)
C2—C3—C4—Cl1	179.0 (3)	C12—C13—C14—N3	−1.2 (4)
C3—C4—C5—C6	0.4 (6)	C14—N3—C15—C20	−173.4 (4)
Cl1—C4—C5—C6	179.7 (3)	N2—N3—C15—C20	6.9 (5)
C2—C1—C6—C5	−2.7 (6)	C14—N3—C15—C16	6.5 (6)
N1—C1—C6—C5	−178.6 (4)	N2—N3—C15—C16	−173.2 (3)
C4—C5—C6—C1	1.8 (6)	C20—C15—C16—C17	−2.4 (6)
C1—N1—C7—C9	168.6 (4)	N3—C15—C16—C17	177.7 (3)
C1—N1—C7—C8	−10.3 (6)	C15—C16—C17—C18	−0.4 (6)
N1—C7—C9—C10	−0.9 (6)	C16—C17—C18—C19	2.7 (6)
C8—C7—C9—C10	178.0 (4)	C16—C17—C18—S1	−177.2 (3)
C7—C9—C10—O1	8.5 (6)	O3—S1—C18—C17	−7.9 (4)
C7—C9—C10—C13	−169.7 (4)	O4—S1—C18—C17	120.6 (3)
N3—N2—C12—C13	−0.8 (4)	N4—S1—C18—C17	−124.2 (3)
N3—N2—C12—C11	176.8 (3)	O3—S1—C18—C19	172.2 (3)
O1—C10—C13—C14	4.9 (6)	O4—S1—C18—C19	−59.2 (4)
C9—C10—C13—C14	−176.9 (4)	N4—S1—C18—C19	56.0 (4)
O1—C10—C13—C12	−173.8 (4)	C17—C18—C19—C20	−2.1 (6)
C9—C10—C13—C12	4.4 (8)	S1—C18—C19—C20	177.7 (3)
N2—C12—C13—C14	1.3 (4)	C18—C19—C20—C15	−0.7 (6)
C11—C12—C13—C14	−176.0 (4)	C16—C15—C20—C19	3.0 (6)
N2—C12—C13—C10	−179.8 (4)	N3—C15—C20—C19	−177.1 (3)

### Hydrogen-bond geometry (Å, °)

**Table:**

*D*—H···*A*	*D*—H	H···*A*	*D*···*A*	*D*—H···*A*
O1—H1O···O2	0.84	1.79	2.498 (4)	141
N1—H1···O1	0.88	2.00	2.659 (4)	131
N1—H1···O3^i^	0.88	2.34	3.093 (4)	143
N4—H41···N2^ii^	0.88	2.16	3.003 (4)	161
N4—H42···O4^iii^	0.88	2.09	2.917 (4)	156

Symmetry codes: (i) −*x*+1, −*y*+1, −*z*+1; (ii) *x*, *y*, *z*−1; (iii) −*x*, −*y*+1, −*z*.
